# Spanish primary students’ writing attitudes and perceived family support: a socioemotional perspective

**DOI:** 10.3389/fpsyg.2026.1754157

**Published:** 2026-02-11

**Authors:** Patricia Robledo, Sara Real Castelao, Celestino Rodríguez, María Lourdes Álvarez-Fernández

**Affiliations:** 1 Department of Psychology, Sociology and Philosophy, Universidad de León, León, Spain; 2 Department of Psychology, University of Oviedo, Oviedo, Spain

**Keywords:** attitude toward writing, cultural context, family support, home literacy environment, primary education, socioemotional learning

## Abstract

Writing is an essential skill for students’ academic, personal, and social development, and learning to write is mediated by the experiences in the family setting. The present study was conducted during the 2024–2025 academic year. This study analyses the home writing practices of primary school students, their perceptions of family support, and their attitudes toward writing, along with the relationships between these variables throughout students’ primary education. A total of 917 children in their 1st–6th years of primary education participated (468 boys and 449 girls). They attended eight different Spanish primary schools. Data were collected using a set of questions created for the study that focused on children’s day-to-day writing practices at home and the “Writing Attitude Scale.” The results show that the most common home writing practices were associated with functional and academic tasks, while expressive practices were less common. This pattern was the same throughout primary schooling, although there were some significant variations by school year. There was a significant progression in the use of digital devices for writing as students advanced in primary school, as well as a fall in family support for writing tasks, particularly in the later years. Nonetheless, students’ affective responses toward writing were stable and mostly positive. Positive correlations were found between the frequency of home writing practices, family support (particularly from mothers), and students’ favorable attitudes toward writing. These findings underscore the importance of the family environment as a key mediating agent in the development of writing skills, and the need to promote active, motivating family involvement throughout primary education.

## 1 Introduction

### 1.1 Writing competence as socio-cultural and motivational process

Writing competence is an essential skill for personal, academic, and professional life. It is a key instrument for human communication, participation, and development. As UNESCO (2019) noted, knowing how to write effectively is a powerful means of expression and learning, and a primary objective of education systems all over the world. Along these lines, the Council of the European Union (2018) and the European Commission (2020) recognize reading and writing as key competencies for permanent learning, involving understanding, creation, and interpretation of ideas, emotions, and values in various formats and contexts, including digital. In a globalized, interconnected world, the ability to produce clear, coherent, suitable texts for a range of communicative purposes is fundamental to having active citizens, employability, and social inclusion. Far from being a merely technical skill, writing is a complex activity that develops within social and cultural contexts and is shaped by the interaction between individual, social, and instructional factors within writing communities. As a social and cultural practice, writing is deeply involved in the construction of knowledge, the expression of identity, and emotional regulation (Bazerman et al., 2018; Graham, 2023; Van Waes and Leijten, 2015).

From this broad perspective, learning to write means joining discursive communities that give meaning to the practice of writing. The Writer(s)-within-Community model (Graham, 2018; Graham, 2023) summarizes this view by conceptualizing writing as a situated social activity, influenced by the writer’s cognitive, emotional, and motivational resources and by the shared norms, values, and purposes of the community in which it is done. These internal resources include executive functions such as working memory, which play a critical role in managing the cognitive demands of writing; recent intervention studies have shown that strengthening working memory updating leads to significant improvements in primary school students’ writing ability and performance (Gao et al., 2023). In line with this view, evidence from primary education indicates that writing performance is shaped by the joint contribution of cognitive and motivational variables (Graham et al., 2019; Rocha et al., 2024). Writers develop within communities in which significant adults—teachers and family members—act as mediators of learning and self-regulation for diverse communicative purposes (Graham, 2018, 2023). Recent research has further emphasized that the quality of adult–child writing interactions, particularly through modeling and scaffolding practices that attend to meaning-making and engagement, plays a central role in early writing development (Bingham and Gerde, 2023). Writing communities thus function as dynamic systems in which individual and social factors are co-determined, and where experiences, available tools, and interpersonal relationships jointly shape the development of writing competence. Within this framework, social contexts such as the family are conceptualized as being closely associated with children’s motivation and attitudes toward writing through the practices, expectations, and support provided by significant adults. This perspective is theoretical and contextual in nature and does not imply the examination of statistical moderation effects in the present study.

### 1.2 Family support, home writing practices, and writing attitudes in primary school

From this perspective, the learners’ environment—both in terms of education and motivation—is key to their progress. In this learning context, the school constitutes the formal setting in which teachers guide the development of writing skills and strategies through structured instructional practices (Freedman et al., 2016; Malpique et al., 2023a) Nonetheless, learning to write transcends the classroom and extends to informal spaces, particularly the home, where families play an essential role by providing support and modeling of writing-related behaviors (Aram and Yashar, 2023).

To ensure conceptual clarity, it is important to distinguish between two related but distinct constructs. The family environment refers to the broader socio-emotional and cultural context of the home in which writing-related opportunities, routines, values, and emotional climates are embedded. By contrast, family participation (or family support) denotes the specific, observable behaviors through which family members actively engage in children’s writing, such as helping with writing tasks, reviewing or correcting texts, or expressing interest in what children write. Family participation can thus be understood as one way in which the family environment is enacted in everyday writing interactions (Aram and Yashar, 2023; Kelso-Marsh et al., 2025).

The family environment is not just a complementary setting to teaching in school, but rather a fundamental setting for children learning to write. Families introduce children to the functional and emotional dimensions of written language, offering day-to-day opportunities for meaningful learning- such as making lists, writing real communicative messages, and participating in fun activities writing activities—and the quality of this support is closely linked to parents’ understanding of how writing develops and how it can be fostered (Aram and Yashar, 2023; Elish-Piper, 2010; Malpique et al., 2023b). The empirical evidence shows that families can offer different forms of support to the process of learning to write. On the one hand, they offer direct instructional help, based on providing guidance on writing skills or strategies that are particularly effective in children’s early schooling (Alston-Abel and Berninger, 2018; Aram et al., 2014; Aram and Yashar, 2023; Gázquez Linares et al., 2020). On the other, they offer motivational support, aimed at strengthening children’s initiative, effort, and self-perceptions of competence as writers, encouraging engagement and enjoyment of the task (Camacho and Alves, 2017; Kelso-Marsh et al., 2025; Robledo and García, 2013). Taken together, these factors make up a literacy environment that can have a positive or negative impact on the process of students developing writing competence.

Several recent studies have shown that students’ attitudes toward writing, and their perceptions of competency are closely related to the emotional and cultural support present in the family environment (Gardner, 2013; Izquierdo-Magaldi et al., 2020; Kelso-Marsh et al., 2025; Robledo Ramón and García Sánchez, 2009). In this regard, the home is not only a functional space for literacy, but also an affective setting shaping children’s beliefs, interests and dispositions toward writing (Kelso-Marsh et al., 2025). In this framework, attitudes toward writing are understood as learned predispositions that include affective, cognitive, and evaluative components (Ekholm et al., 2018; Alves-Wold et al., 2023, 2024), and which largely determine students’ levels of engagement with writing tasks. The empirical evidence suggests that positive attitudes toward writing are usually associated with greater enjoyment and more sustained engagement with composition tasks, as well as better results in terms of text quality. In contrast, negative attitudes are linked to lower motivation, avoidance behaviors, and lower levels of self-efficacy (Bruning and Kauffman, 2016; Graham et al., 2017; Skar et al., 2023). Therefore, parents’ participation in students learning to write cannot only facilitate development of instrumental skills but also encourage formation of positive attitudes and sustained intrinsic motivation for writing.

### 1.3 Research gap and contribution of the present child-reported study

Despite this, family participation—understood as the set of concrete supportive behaviors through which the family environment is enacted in everyday writing interactions—in childrens learning to write is an area that has received limited empirical attention (Alston-Abel and Berninger, 2018; DeFauw, 2017; Kelso-Marsh et al., 2025). The family environment constitutes a multidimensional construct that encompasses a range of interrelated factors, including parental instructional and motivational support, affective climate, parental beliefs and attitudes toward writing, and broader family literacy resources, all of which have been shown to influence children’s writing practices and motivation to varying degrees (Aram and Yashar, 2023; Graham, 2018; Kelso-Marsh et al., 2025) Most existing studies have concentrated on early childhood education or the initial years of primary school, while comparatively fewer have examined the role of the family setting throughout primary education—a critical stage in which writing instruction becomes progressively more formal and systematic (Malpique et al., 2023a). This becomes particularly important if we bear in mind that motivation and attitudes toward writing tend to fall progressively throughout primary education (Marbà and Márquez, 2010; National Literacy Trust, 2023, 2024; Regueiro et al., 2015). Understanding how the home environment is associated with children’s interest, emotional engagement, and attitudes toward writing at this stage is therefore essential for advancing knowledge about writing development during primary education.

In addition, previous methodological approaches have suffered from notable limitations. In most cases, knowledge about the home writing environment has been obtained solely through questionnaires completed by parents, which may have introduced biases related to social desirability, and has often left children without a direct means of voicing their own experiences (Gardner, 2013; Kelso-Marsh et al., 2025; Malpique et al., 2023a).

By contrast, collecting data directly from children makes it possible to access their own perceptions of family support and writing experiences, which is particularly relevant when examining attitudes toward writing and affective responses to writing tasks. This child-reported perspective reduces reliance on adult informants and provides a more proximal account of the socio-emotional processes involved in learning to write, which may not be fully captured through parent-reported measures alone (Gardner, 2013; Graham, 2018, 2023)

Only a single study, by Gardner (2013), has directly addressed the perspective of 6–9-year-old students, identified by their teachers as “reluctant writers,” combining teacher interviews with structured and semi-structured questionnaires aimed at the children. That analysis showed a marked discontinuity between home and school writing practices. Although in school children exhibited low motivation and negative attitudes toward writing, in the home they described greater engagement in various types of writing activities, accompanied by emotional and practical support with real family audiences who lent legitimacy to what they produced. This contextual difference suggests that the family constitutes a relevant contextual framework in which children’s writing attitudes and perceptions of competence are experienced and shaped, potentially supporting motivation and enjoyment in ways that complement formal school instruction, without implying the examination of statistical moderation effects in the present study. However, the scope is restricted to a limited age group and a specific student group, which underscores the need to expand research to other ages and student profiles in order to understand the range of experiences and attitudes toward writing throughout primary education.

In this area, there is still a relevant gap in our knowledge regarding how children perceive family support for learning to write and how these perceptions are related to their attitudes toward writing across primary education. Recent research has described family support and home writing practices primarily from an adult-reported perspective, focusing on parents’ accounts of involvement and support strategies (Algorri-Diez et al., 2025). By contrast, the present study adopts a *child-centered approach*, understood as an educational perspective that prioritizes children’s agency, interests, emotional engagement, and active participation in meaningful writing experiences, and that gives direct voice to students’ own perceptions. Recent research indicates that such child-centered approaches are particularly relevant for understanding motivation and attitudes toward writing when literacy experiences are embedded in supportive social interactions (Skar et al., 2023). Accordingly, this study examines children’s perceptions of family support for writing and analyses how these perceptions are associated with writing attitudes and home writing practices. Addressing this dimension is essential for advancing the understanding of the socio-emotional role of the home environment in the development of writing competence and for refining explanatory models grounded in students’ livedexperiences.

### 1.4 Objectives and hypotheses

This is the context of the present study, whose overall objective is to identify the home writing practices of students in the 1st–6th years of primary school, as well as their perceptions and assessments of the family support they get, and the relationship to their attitudes toward writing.

The study has three specific objectives. First, to analyze the home writing practices that primary school students say that they do and how those practices change during primary education. Second, to analyze children’s perceptions of family support in writing tasks and their affective responses toward this support across primary education. Lastly, to explore the relationship between home writing practices, perceived family support, and children’s attitudes toward writing.

Based on these objectives and previous research, the following hypotheses were formulated.

*H1*: Functional or academic writing activities (e.g., schoolwork, lists, short messages) will be more frequent in the home context than expressive or creative writing practices (e.g., journals, stories, poems) (Gardner, 2013; Malpique et al., 2023a).

*H2*: The frequency of formal writing practices at home will increase as students progress through primary education, reflecting greater alignment with school-related writing demands over time (Gardner, 2013; Malpique et al., 2023a).

*H3*: Perceived family support for writing will be higher in the early years of primary education, reflecting greater adult involvement at initial stages of writing development (Aram et al., 2014). Recent research suggests that family support may vary in both nature and intensity across primary education cycles, indicating qualitative changes rather than a strictly linear pattern of decline (Algorri-Diez et al., 2025).

*H4*: Perceived family support for writing will tend to decrease as students gain autonomy across school years, in line with developmental changes in adult involvement in writing activities (Aram et al., 2014; Bingham and Gerde, 2023).

*H5:* Mothers will be identified as the primary providers of writing support in the home context (Aram, 2010; Algorri-Diez et al., 2025).

*H6*: Regardless of school year, children will tend to report predominantly positive emotional responses to family writing support, reflecting the affective value of supportive writing interactions (Graham, 2018, 2023).

*H7:* Family writing interactions that include an affective component (e.g., encouragement or emotional support) will be associated with more positive emotional responses to writing activities (Alston-Abel and Berninger, 2018; Robledo and Olivares, 2022).

*H8*: Family writing interactions that include a communicative component (e.g., showing interest in or discussing children’s texts) will be associated with more positive emotional responses to writing activities (Alston-Abel and Berninger, 2018; Bingham and Gerde, 2023; Graham, 2018).

*H9*: Higher levels of perceived family support and greater engagement in home writing activities will be associated with more favorable attitudes toward writing, whereas limited meaningful support or writing experiences will be associated with less positive attitudes (Camacho and Alves, 2017; Newman and Bizzarri, 2011; Robledo and García, 2013; Rasteiro and Limpo, 2023).

## 2 Methodology

### 2.1 Sample

Convenience sampling was used, with participating students being selected on the basis of accessibility provided by the collaborating schools in Spain. This approach is commonly used in school-based research when access depends on institutional collaboration; however, it requires transparent reporting and cautious interpretation regarding generalizability because nonprobability samples may introduce selection bias (Savitsky et al., 2023; Stratton, 2023; Zickar and Keith, 2023) The type of schools and geographical location were considered. The final sample was made up of eight schools, six publicly funded and two semi-private schools, three of which were in rural locations, and five in urban settings. This allowed a heterogeneous sample to be produced that is representative of the diversity in primary education.

In total, 917 primary school students participated, 468 were boys, 449 were girls. They were aged between 6 and 13 years old (*M* = 8.70; SD = 1.69). This age range is particularly important, as it is the time period in which formal teaching of writing is consolidated and attitudes toward writing become established (Graham et al., 2017; Rasteiro and Limpo, 2023; Skar et al., 2023). Table 1 shows the detailed distribution of students by school year and sex.

**TABLE 1:** Sample distribution by sex and school year.

Sex	1st	2nd	3rd	4th	5th	6th	Total
Girl	69	68	63	70	95	84	449
Boy	90	86	54	77	87	74	468
Total	159	154	117	147	182	158	917

Prior to data collection, written informed consent was obtained from the students’ families or legal guardians after they had been informed about the study objectives, procedures, voluntary nature of participation, and data confidentiality. Only students whose families provided informed consent participated in the study.

### 2.2 Measures

Two instruments were used to evaluate the main study variables—home writing practices, perceived family support, and attitudes toward writing. The first comprises specific questions about everyday writing at home, designed to describe the home writing context and to characterize the different types of family support perceived by students. The second, a standard questionnaire that has been widely validated in the international literature, evaluates students’ overall attitudes toward writing, including affective and motivational components and enjoyment.

Questions about everyday writing at home (Table 2). Fifteen questions were selected by a team of experts in writing and family literacy, using the questions from the Gardner (2013) study as a reference. The instrument is organized into two sections. The first (questions 1–12) identifies the writing activities that students carry out at home, classified as formal practices (related to the academic domain) and informal practices (spontaneous or functional), including digital writing practices. Each question assesses whether these practices are present or absent.

**TABLE 2:** Primary school students’ reported home writing practices: adapted from Gardner (2013) .

Item content	
**At home I do these writing activities, on my own or with someone?**	
1	I write lists (shopping lists, guest lists, etc.)
2	I write short notes or messages
3	I write letters, cards, or invitations
4	I write songs, jokes, poems, or riddles
5	I write in a diary or journal
6	I write stories
7	I write letters, words, sentences, or texts for fun
8	I practice handwriting (to improve it)
9	I practice spelling (to avoid mistakes)
10	I do school writing tasks
11	I write messages on WhatsApp, email, or social networks
12	I write on a tablet, computer, or mobile phone
**At home, who …?**	
13	Helps me when I do writing activities. Do you like this help? (Yes/No/It’s OK)
14	Reviews or corrects what I write. Do you like this help? (Yes/No/It’s OK)
15	Pays attention to what I write (reads it, shows it to others, congratulates me, etc.). Do you like this help? (Yes/No/It’s OK)

The second section (questions 13–15) explores the existence and nature of family support for writing tasks, as well as who provides that support. Students indicate which family member provides three possible types of help: (a) direct support in carrying out writing tasks; (b) review or correction of texts; and (c) attention to and interest in what they write, accompanied by positive reinforcement. In addition, an evaluative item is included (“Do you like this help?”), where the child can express their emotional assessment by choosing one of three responses (“yes,” “it’s ok,” or “no”) for each type of support.

The Writing Attitude Scale (Graham et al., 2017). A self-report instrument with 5 items, each using a five-point Likert response (1–5) assessing overall enjoyment and assessment of writing in school and non-school contexts. Higher scores indicate more positive attitudes. It has a unifactorial structure and high internal consistency in primary school children and students in early secondary education: α = 0.83–0.96); more specifically, α = 0.83 in primary students (Graham et al., 2017), α = 0.96 in 5th grade primary (Graham et al., 2019) and α = 0.91/0.88 in 6th–7th grade (Rasteiro and Limpo, 2023). There is also validation evidence in Portuguese which maintains the 5-item format and the unidimensionality (Rocha et al., 2024).

### 2.3 Design and procedure

The study followed a quantitative, descriptive, comparative design framework, and was transversal. The first step was a thorough theoretical-empirical review to provide a conceptual basis for the study and to outline the study variables. Following that, the evaluation instruments were designed and selected, in line with the study objectives.

The study was conducted during the 2024–2025 academic year. Preparatory activities began in September 2024 and included the selection of participating schools, the acquisition of institutional permissions, and meetings with school principals. Data collection was carried out between February and June 2025, coinciding with the regular school calendar, once ethical approval and written informed consent from families had been obtained.

The study was approved by the Ethics Committee of the University of León (Spain, code ETICA-ULE-026-2024), within the framework of Project PID2021-1244011-NB-I00, funded by the Spanish Ministry of Science and Innovation.

The study was carried out in conformance with national and European data protection legislation, in accordance with European Parliament and Council of the European Union (2016) European Parliament regulation 2016/679 of the 27th April 2016, related to protection of personal data and the movement of such data, and with Agencia Estatal Boletín Oficial del Estado (2018) Organic Law 3/2018, 5th December, on personal data protection and guarantee of digital rights.

Once approval had been obtained from the ethics committee, contact was made with the schools in the province, aiming for balanced representation of public and independent (and rural or urban) schools. Once schools agreed to participate, the school management informed the families about the study objectives and the data collection procedures. Only students whose families gave their informed consent were included in the sample.

The evaluations were done on paper in class groups, during normal teaching hours. Two trained evaluators participated in each session. Their role was to give clear, consistent instructions, to ensure that all of the students understood what they had to do and answer any questions. To ensure understanding, the evaluators explained the purpose of each instrument, gave examples, and read out each item, making sure the students understood the questions/items before responding.

Following the field work, the materials were collected and coded in a database in Microsoft Excel. The data were then analyzed using SPSS, version 30 for Windows (IBM Corp, 2024).

### 2.4 Data analysis

Three blocks of analysis were conducted in line with the study objectives. First, descriptive statistics (frequencies and percentages) were calculated to characterize home writing practices (Objective 1). This approach is appropriate for summarizing patterns of writing activities and perceived family support in large samples and has been used in recent survey-based research on writing instruction and practices across primary education (Malpique et al., 2023a).

Second, multivariate analyses of variance (MANOVAs) were conducted to examine differences across school years (1st–6th grade), with home writing practices, perceived family support, and students’ affective responses as dependent variables (Objective 2). MANOVA was selected due to the multivariate nature of the dependent variables and to control for Type I error when comparing multiple related outcomes, following recent educational and language research adopting similar analytical strategies (Malpique et al., 2023a; Lira-Gonzales et al., 2024). Levene’s test of equality of variances indicated violations of the homogeneity assumption for several dependent variables (see Table 3). Consequently, Pillai’s trace was interpreted in the MANOVA, as it is considered the most robust multivariate statistic when assumptions are violated. When significant multivariate effects were detected, Welch’s ANOVAs were conducted for univariate comparisons, followed by Games–Howell *post-hoc* tests, which do not assume equal variances or equal sample sizes.

**TABLE 3:** Homogeneity of variances.

Variables	Levene	gl1	gl2	*p*
Attitude 1	0.874	5	914	0.498
Attitude 2	1,298	5	913	0.263
Attitude 3	1,949	5	914	0.084
Attitude 4	1,816	5	913	0.107
Attitude 5	1,609	5	911	0.155
Attitude tot.	2,185	5	905	0.054
I write lists (shopping lists, guest lists, etc.)	8,356	5	897	< 0.001
I write short notes or messages	39,791	5	887	< 0.001
I write letters, cards, or invitations	6,184	5	879	< 0.001
I write songs, jokes, poems, or riddles	2,020	5	879	0.074
I write in a diary or journal	5,195	5	894	<0.001
I write stories	7,580	5	884	<0.001
I write letters, words, sentences, or texts for fun	8,256	5	894	<0.001
I practice handwriting (to improve it)	4,616	5	896	<0.001
I practice spelling (to avoid mistakes)	1,445	5	881	0.206
I do school writing tasks	54,963	5	886	<0.001
I write messages on WhatsApp, email, or social networks	13,673	5	888	<0.001
I write on a tablet, computer, or mobile phone	46,096	5	891	< 0.001
Total	5,103	5	892	<0.001
At home, my mother helps me when I do writing activities	14,034	5	908	<0.001
At home, my father helps me when I do writing activities	41,534	5	909	< 0.001
At home, others help me when I do writing activities	8,233	5	909	< 0.001
At home, no-one helps me when I do writing activities	29,857	5	909	< 0.001
At home, my mother reviews or corrects what I write	13,077	5	908	< 0.001
At home, my father reviews or corrects what I write	27,485	5	908	< 0.001
At home, others review or correct what I write	3,424	5	908	0.005
At home, no-one reviews or corrects what I write	52,412	5	906	< 0.001
At home, my mother pays attention to what I write	7,335	5	908	< 0.001
At home, my father pays attention to what I write	1,499	5	908	0.188
At home, others pay attention to what I write	9,030	5	908	< 0.001
At home, no-one pays attention to what I write	18,877	5	907	< 0.001
13. Do you like this help?	7,448	5	639	< 0.001
14. Do you like this help?	5,525	5	696	< 0.001
15. Do you like this help?	4,957	5	741	< 0.001

Finally, Pearson correlation analyses were conducted to examine the relationships between home writing practices, perceived family support, and attitudes toward writing (Objective 3). Pearson correlations are appropriate for assessing associations between continuous variables and have been widely used in recent studies examining links between writing performance, motivational variables, and contextual factors (Rocha et al., 2024).

## 3 Results

### 3.1 Primary school students’ home writing practices

#### 3.1.1 Frequency of students’ home writing practices

**FIGURE 1:**
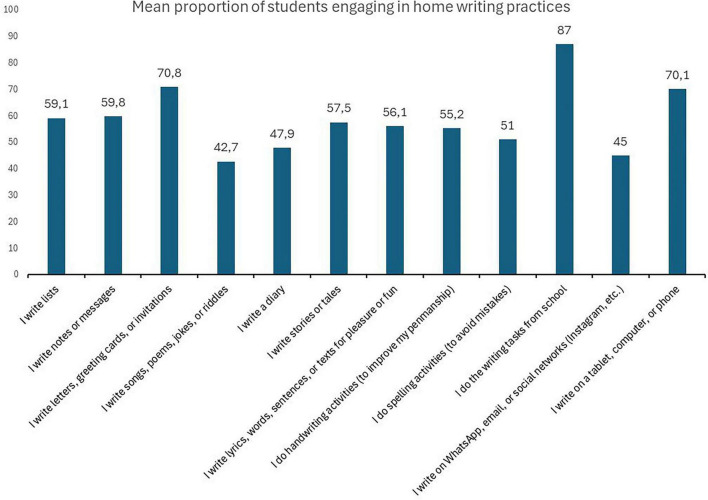
Frequency of home writing practices done by primary school students. The figure shows the percentage of students who indicated doing each writing activity at home, either on their own or with family support.

The first objective of this study was to descriptively analyze the home writing practices of primary school students. As Figure 1 shows, the most common activities were related to schoolwork, the use of digital devices, and writing cards, greetings, or invitations. In addition, there was a substantial presence of functional practices, such as writing lists or short notes, which shows how writing was incorporated into daily home life. In contrast, expressive or creative practices such as writing stories, poems and songs, or keeping a personal diary or journal, were much less common, suggesting that this more personal, playful dimension of writing was present in the family environment in a more limited manner.

#### 3.1.2 Progression of primary school students’ home writing practice by school year

A MANOVA was conducted to examine differences across school years. Given the violation of the homogeneity of variances assumption and the sensitivity of Box’s test (*M* = 900.631, *p* < 0.001), Pillai’s trace was used. The MANOVA analyses demonstrated statistically significant effects of school year, with a moderate effect size [Pillai = 0.336, *F*(60, 3880) = 4.663, *p* < 0.001, η^2^ = 0.067]. Subsequent univariate analyses were performed using Welch’s ANOVA, with Games–Howell *post hoc* comparisons. The ANOVA indicated significant differences between school years in the variables shown in Table 4, confirming the influence of school year on the frequency of different home writing practices.

**TABLE 4:** Results of ANOVA with Welch corrections for home writing practices by school year.

Variables	1st		2nd		3rd		4th		5th		6th		ANOVA			Welch			
	*M*	SD	*M*	SD	*M*	SD	*M*	SD	*M*	SD	*M*	SD	*F*	*p*	η ^2^	Welch	gl1	gl2	*p*
1. I write lists (shopping lists, guest lists. etc.)	0.53	0.50	0.53	0.50	0.63	0.48	0.64	0.48	0.65	0.47	0.57	0.49	3.108	0.009	0.017	3.099	5	409.002	0.009
2. I write short notes or messages	0.54	0.501	0.55	0.500	0.57	0.498	0.56	0.498	0.61	0.490	0.77	0.425	5.694	< 0.001	0.031	6.804	5	400.485	< 0.001
5. I write in a diary or journal	0.51	0.502	0.54	0.500	0.58	0.496	0.44	0.498	0.46	0.500	0.37	0.483	4.700	< 0.001	0.026	4.802	5	406.059	< 0.001
6. I write stories	0.62	0.487	0.59	0.494	0.69	0.466	0.55	0.500	0.55	0.499	0.48	0.501	2.831	0.015	0.016	2.847	5	400.194	0.015
7. I write letters, words, sentences, or texts for fun	0.66	0.475	0.50	0.502	0.66	0.478	0.51	0.502	0.53	0.500	0.57	0.496	3.105	0.009	0.017	3.135	5	406.146	0.009
8. I practice handwriting (to improve it)	0.66	0.475	0.61	0.489	0.59	0.494	0.57	0.497	0.55	0.499	0.33	0.472	8.247	< 0.001	0.044	8.600	5	406.788	< 0.001
9. I practice spelling (to avoid mistakes)	0.54	0.50	0.47	0.50	0.56	0.49	0.51	0.50	0.56	0.49	0.42	0.49	2.378	0.037	0.013	2.387	5	398.854	0.038
10. I do school writing tasks	0.72	0.449	0.87	0.337	0.85	0.360	0.92	0.275	0.95	0.228	0.95	0.215	13.383	< 0.001	0.070	10.687	5	391.360	< 0.001
11. I write on WhatsApp, Email, social networks	0.37	0.485	0.23	0.420	0.39	1.011	0.40	0.492	0.51	0.501	0.81	0.391	21.842	< 0.001	0.110	39.431	5	394.515	< 0.001
12.I write on a Tablet, Computer, mobile phone, etc.	0.43	0.496	0.55	0.500	0.72	0.993	0.78	0.417	0.83	0.377	0.97	0.202	23.364	< 0.001	0.116	46.358	5	383.563	< 0.001
Total	6.73	3.056	6.45	2.493	7.41	2.950	7.01	2.391	7.35	2.249	7.39	1.905	4.804	< 0.001	0.026	5.038	5	400.565	< 0.001

Practices related to using digital technologies, such as *writing on social networks or messaging applications* and *writing on digital devices* exhibited moderate effect sizes, demonstrating substantial variation by school year. The means values (Table 5) show a progressive increase in the frequency of these activities over the course of primary schooling.

**TABLE 5:** *Post-hoc* tests on the means for home practices.

Variables	Course		*P*
1.I write lists	2nd	3rd	0.027
2. I write short notes or messages	6th	1st	<0.001
		2nd	<0.001
		3rd	<0.001
		4th	0.002
		5th	0.018
5. I write in a diary or journal	6th	2nd	0.009
		3rd	<0.001
	3rd	4th	0.034
		5th	0.037
6. I write stories	6th	3rd	0.017
7. I write letters, words, sentences, or texts for fun	2nd	1st	0.048
		3rd	0.031
8. I practice handwriting (to improve it)	6th	1st	<0.001
		2nd	<0.001
		3rd	<0.001
		4th	<0.001
		5th	<0.001
10. I do school writing tasks	1st	2nd	0.013
		4th	<0.001
		5th	<0.001
		6th	<0.001
	3rd	5th	0.007
		6th	0.006
11. I write WhatsApp, email, on social networks	1st	2nd	0.029
		5th	0.049
		6th	<0.001
	2nd	4th	0.007
		5th	<0.001
		6th	<0.001
	6th	3rd	<0.001
		4th	<0.001
		5th	<0.001
12. I write on a tablet, computer, mobile phone, etc.	1st	4th	<0.001
		5th	<0.001
		6th	<0.001
	2nd	4th	<0.001
		5th	<0.001
		6th	<0.001
	6th	3rd	0.037
		4th	<0.001
		5th	<0.001

The variables *doing school writing tasks, handwriting and spelling activities* exhibited moderate and small effect sizes.

The remaining practices, such as *writing notes or short messages, keeping a diary/journal, writing stories*, and *writing for pleasure or fun*, exhibited small, albeit statistically significant effects, indicating smaller differences between educational levels.

Finally, the total amount of home writing practices varied significantly by school year, with a small effect (η^2^ = 0.026).

*Post hoc* analysis (Games–Howell) showed statistically significant differences between school years in various writing practices (Table 5). In the activity, “I write list” the students in 2nd grade differed significantly from 3rd grade students, who had higher scores. In “I write short notes or messages at home,” the students in 1st, 2nd, and 4th grade differed significantly from 6th grade students, with the latter scoring higher. In “I write a diary” the students in 2nd–3rd grade differed significantly from 4th, 5, 6th grade students (4th–6th lower scores). In activity “I write stories” the students in 3rd grade differed significantly from 6th grade students, who had lower scores, In “Write for fun” the students in 1st and 3rd grade differed significantly from 2nd grade students, who had lower scores. In the variable, “I do handwriting activities,” there were differences between students in grades 1–5 compared to the 6th graders, who had lower scores. In, “I do school writing tasks,” 1st graders scored significantly lower than students in 2nd, 4th, 5th, and 6th grade; finally 3rd grades scored significantly lower than students in 5–6th grade. In the activity, “I write on social networks, WhatsApp, etc.,” 2nd graders’ responses differed from 1st, 4th, and 5th graders’, and 6th graders differed from those in grades 1–5, with higher scores in the later school years and lower scores in 2nd. Similarly, in, “I write on a tablet, computer, or mobile,” there were significant differences between 1st and 2nd graders and those in grades 4, 5, and 6, and between 6th graders and 3rd, 4th, 5th graders, with higher scores in the later school years.

### 3.2 Students’ perceived family support for writing activities

Given the violation of the homogeneity of variances assumption and the sensitivity of Box’s test (Box’s *M* = 741.321, *p* < 0.001), a robust multivariate approach was adopted, using Pillai’s trace. The MANOVA revealed statistically significant effects of school year on the variables examined, with a small effect size [Pillai = 0.186, *F*(60, 4480) = 2.885, *p* < 0.001, η^2^ = 0.037]. Follow-up analyses using Welch’s ANOVAs indicated significant differences between school years in several of the variables analyzed (Table 6), related to the support, correction, and attention associated with writing in the family context, as well as to the family member providing such support. *Post hoc* comparisons (Games–Howell) further confirmed these differences across school years (Table 7).

**TABLE 6:** Results of ANOVA with Welch corrections related to perceived help, by school year.

Variables	1st		2nd		3rd		4th		5th		6th		ANOVA			Welch			
	M	SD	M	SD	M	SD	M	SD	M	SD	M	SD	F	p	η ^ 2^	Welch	gl1	gl2	*p*
13. At home, my mother helps me when I do writing activities	0.78	0.420	.69	0.458	0.74	0.441	0.70	0.460	0.65	0.479	0.54	0.500	4.814	< 0.001	0.026	4.645	5	412.771	< 0.001
13. At home, my father helps me when I do writing activities	0.50	0.502	0.49	0.502	0.48	0.502	0.52	0.501	0.36	0.483	0.23	0.424	8.143	< 0.001	0.043	9.382	5	411.444	< 0.001
13. At home, no-one helps me when I do writing activities	0.14	0.353	0.18	0.389	0.13	0.338	0.22	0.414	0.31	0.464	0.37	0.485	8.046	< 0.001	0.042	7.630	5	415.657	< 0.001
14. At home, my mother reviews or corrects what I write	0.73	0.446	0.77	0.422	0.79	0.408	0.72	0.450	0.67	0.470	0.57	0.496	4.397	< 0.001	0.024	4.111	5	413.911	< 0.001
14. At home, my father reviews or corrects what I write	0.52	0.501	0.48	0.501	0.54	0.501	0.48	0.501	0.37	0.484	0.26	0.443	6.784	< 0.001	0.036	7.412	5	411.256	< 0.001
14. At home, no-one reviews or corrects what I write	0.07	0.255	0.09	0.281	0.04	0.205	0.13	0.337	0.22	0.416	0.30	0.458	12.340	< 0.001	0.064	11.175	5	417.977	< 0.001
15. At home, others pay attention to what I write	0.22	0.416	0.27	0.445	0.30	0.458	0.29	0.453	0.39	0.490	0.31	0.464	2.636	0.022	0.014	2.555	5	412.221	0.027
15. At home, no-one pays attention to what I write	0.08	0.265	0.17	0.378	0.10	0.307	0.20	0.404	0.19	0.396	0.23	0.419	3.959	0.001	0.021	5.106	5	411.501	< 0.001

**TABLE 7:** *Post-hoc* tests related to home support.

Variables	Year		*P*
**13. At home, my mother helps me when I do writing activities**	6th	1st	0.001
		3rd	0.009
		4th	0.049
13. At home, my father helps me when I do writing activities	6th	1st	<0.001
		2nd	<0.001
		3rd	<0.001
		4th	<0.001
13. At home, my no-one helps me when I do writing activities	1st	5th	0.003
		6th	<0.001
	2nd	6th	0.003
	3rd	5th	0.003
		6th	<001
	4th	6th	<001
14. At home, my mother reviews or corrects what I write	6th	1st	0.035
		2nd	0.003
		3rd	<0.001
14. At home, my father reviews or corrects what I write	6th	1st	<0.001
		2nd	<0.001
		3rd	<0.001
		4th	0.002
14. At home, my no-one reviews what I write	1st	5th	<0.001
		6th	<0.001
	2th	5th	0.006
		6th	<0.001
	3rd	5th	<0.001
		6th	<0.001
	4th	6th	0.004
15. At home, others pay attention to what I write	1st	5th	0.008
15. At home, no-one pays attention to what I write	1st	4th	0.014
		5th	0.015
		6th	0.002

In the variable, “Who helps me when I do writing activities?”, there were significant differences in maternal help between 6th graders and those in grades 1, 3, and 4 courses, with lower scores from the older children. There was a similar pattern for paternal help, with 6th graders differing significantly from those in grades 1–4. In both cases the frequency of support dropped as students advanced through primary school. In contrast, the option, “nobody helps me” exhibited significant differences in the opposite direction. Students in 5th and 6th grade reported not getting help more often than those in grades 1–4.

In the variable, “Who reviews or corrects what I write?”, there were significant differences between 6th graders and those in grades 1, 2, and 3 when the correction was done by mothers, with lower scores from 6th graders. Similarly, when it was the father, there were differences between 6th graders and those in grades 1–4. In contrast, the option, “nobody reviews what I write” exhibited significant differences in the opposite direction. Students in 5th and 6th grade reported not getting review more often than those in grades 1, 2, and 4.

In the variable, “Who pays attention to what I write?”, the differences were mainly when the response was “others,” which was more common in 1st grade than in 5th. In the option “no-one,” it was 1st graders who differed significantly from 4th, 5th, 6th graders, with a greater proportion of the older students stating that they did not get attention from the family.

Lastly, analysis of the students’ affective responses to the help with home writing tasks (*Do you like this help?*) did not show any significant differences by school year [Pillai = 0.028, *F*_(15, 1458)_ = 0.925, *p* = 0.536, η^2^ = 0.009]. The scores were consistently positive in all levels, with means that were close to the scale maximum (2). The least appreciated type of help throughout primary education was families reviewing or correcting texts.

### 3.3 Relationship between writing practices, family support, and attitudes

The Pearson correlation analysis demonstrated a small, positive, statistically significant correlation [*r*(915) = 0.254, *p* < 0.001] between attitudes toward writing and home writing practices. In addition, there were significant, positive correlations between support offered by mothers and overall attitudes toward writing. More specifically, there were associations between help with writing activities [*r*(915) = 0.140, *p* < 0.001], correction or review [*r*(915) = 0.122, *p* < 0.001], and attention or appreciation of writing [*r*(915) = 0.116, *p* = 0.001].

In line with that, the support offered by fathers was also positively, and statistically significantly correlated with attitudes toward writing: help with writing activities [*r*(915) = 0.114, *p* < 0.001], correction or review [*r*(915) = 0.138, *p* < 0.001], and attention or appreciation of writing [*r*(915) = 0.086, *p* = 0.010].

On the whole, the data showed positive, albeit small, associations between the frequency of home writing practices, perceived family support, and primary school students’ attitudes toward writing.

## 4 Discussion

This study examined primary school students’ home writing practices, their perceptions of family support, and their attitudes toward writing, from the perspective of the students themselves. From socio-cultural and socio-cognitive models of literacy—which emphasize the influence of context and of significant adults in constructing writing competence (Aram et al., 2014; Elish-Piper, 2010; Graham, 2018; Robledo and Olivares, 2022)—this study expands the empirical evidence on the influence of the family environment with a developmental and ecological perspective.

In terms of the first objective, focused on identifying the students’ home writing practices, the results show that functional and academic activities predominated, such as doing schoolwork and writing lists or short messages, while the creative or expressive practices such as writing stories, poems, or diary entries were less common. This pattern is consistent with previous studies describing utilitarian use of writing in the domestic environment (Gardner, 2013; Malpique et al., 2023a). In addition, the results indicate progressively greater use of digital devices to write as children advance through primary school, reflecting the incorporation of new forms of communication and digital literacy in the home (National Literacy Trust, 2023, 2024). In addition, as students progress through primary school, fewer practices focused on mechanical skills such as handwriting are carried out at home (Malpique et al., 2023b).

With regard to the second objective—analyzing the children’s perceptions of family support for writing tasks—we found that mothers were the main source of support, ahead of fathers, replicating patterns identified in previous educational stages (Aram, 2010; Kelso-Marsh et al., 2025). However, the frequency of support dropped significantly in later school years, particularly in 6th grade, where a greater number of students indicated not having support at home. This fall in support seems to be associated with the development of writing autonomy and the parental role moving toward more general supervision. Despite the fall, the students’ affective responses to family help continued to be positive and were stable throughout primary schooling, which confirms that students appreciate their families’ involvement in the writing process, including when it is limited to interest in or appreciation of what they produce. These findings are consistent with Gardner (2013) and align with recent evidence showing positive associations between parental involvement and children’s writing outcomes and attitudes (Kelso-Marsh et al., 2025).

From a developmental perspective, the variation in the strength of associations across school years suggests that the influence of family support on writing attitudes evolves throughout primary education. In the early grades, family involvement tends to be more frequent and more closely tied to children’s motivation and engagement, whereas in later grades its influence appears to weaken as students gain autonomy and writing instruction becomes increasingly formalized (Graham, 2018; Malpique et al., 2023a; Kelso-Marsh et al., 2025). This developmental shift may help explain why students in upper primary grades benefit less from certain forms of direct or corrective support, which may be perceived as less necessary or less congruent with their growing sense of competence.

Differences observed between maternal and paternal support further suggest differentiated roles within the family writing context. Recent research indicates that mothers tend to assume a more central and sustained role in supporting children’s writing activities, while fathers’ involvement is often less frequent and more context-specific, which may account for the distinct associations observed between support from mothers versus fathers and writing attitudes (Kelso-Marsh et al., 2025; Alves-Wold et al., 2024). Importantly, affective and communicative forms of support—such as showing interest in children’s writing or valuing what they produce—appear to play a particularly relevant role in sustaining positive writing attitudes across primary education, even as direct instructional support declines (Alves-Wold et al., 2024; Camacho and Alves, 2017; Graham, 2023).

The third objective was to examine the relationship between home writing practices, perceived family support, and children’s attitudes toward writing. Although the family environment is conceptualized as a potential moderating context within the theoretical framework, the present study did not aim to test statistical moderation effects. Instead, it focused on identifying associations between home writing practices, perceived family support, and writing attitudes from the child’s perspective, providing an empirical basis for future research specifically designed to examine moderation processes. The correlational analysis demonstrated positive, albeit small associations between the frequency of writing practices, parental support, and favorable attitudes toward writing. Support from both mothers and fathers, in the help, correction, and attention dimensions, were significantly related to students’ positive appreciation of writing. These results are consistent with previous studies that have linked family involvement with greater motivation and enjoyment of writing activities (Alves-Wold et al., 2024; Camacho and Alves, 2017; Kelso-Marsh et al., 2025; Robledo and García, 2013).

From a theoretical perspective, our findings contribute to expanding the Writer(s)-within-Community model (Graham, 2023), showing that the home acts as a writing practice community in which family interactions affect the emotional, motivational, and social dimensions of learning to write. In this regard, families provide not only instrumental support, but also create an emotional, symbolic context that influences the construction of the children’s attitudes and writing identities.

Despite our results being consistent with the literature, certain limitations do need to be considered. Using self-reporting instruments could have introduced social desirability biases. Finally, it is worth noting that although the sample was large, it was obtained through convenience sampling, and limited to a single Spanish region, which limits how generalizable the findings are.

Future studies should include samples from other regions and sociocultural contexts; they should include the families’ perspectives and use mixed methodologies that will allow information to be triangulated with direct observation of family interactions. It would also be useful to analyze complementary variables, such as socio-economic level, family structure, self-efficacy, and academic performance, which may influence the practices and support in the home.

## 5 Conclusion

This study examined home writing practices, perceived family support, and writing attitudes among primary school students from 1st to 6th grade, with the aim of examining nine hypotheses derived from previous research and from a child-centered perspective on writing development.

With regard to home writing practices, the results supported Hypothesis 1, as functional or academic activities (e.g., schoolwork, lists, short messages) were reported more frequently than expressive or creative practices (e.g., stories, poems, diaries). In addition, Hypothesis 2 was confirmed, since formal writing practices related to school tasks became increasingly predominant as students progressed through primary education.

Concerning family support, the findings were consistent with the proposed developmental patterns. Hypothesis 3 was supported, as perceived family support for writing was more intense in the early years of primary education. Complementarily, Hypothesis 4 was also confirmed, showing a progressive decrease in family support as students gained autonomy across school years. Regarding the sources of support, Hypothesis 5 was supported, with mothers being identified as the primary providers of writing support throughout primary education.

In line with Hypothesis 6, children reported generally positive emotional responses to family writing support regardless of school year. Moreover, emotional responses were particularly positive when family interactions included an affective component (Hypothesis 7) and when they included a communicative component, such as showing interest in or valuing what children wrote (Hypothesis 8).

Finally, the results supported Hypothesis 9, concerning the relationship between family context and writing attitudes. Higher levels of perceived family support and greater engagement in home writing activities were associated with more favorable attitudes toward writing, whereas limited meaningful writing experiences or reduced family support were associated with less positive attitudes.

Overall, these findings highlight the central role of the family as a socio-emotional context in the development of writing competence and in shaping children’s attitudes toward writing throughout primary education. Families who provide opportunities for meaningful writing, emotional support, and communicative engagement contribute to sustaining children’s motivation and enjoyment of writing, even as direct instructional support declines with age.

Taken together, and from a child-centered perspective, this study extends previous research by providing a more nuanced understanding of how home writing practices and perceived family support relate to children’s writing attitudes across developmental stages, based on students’ own reported experiences.

## Data Availability

The original contributions presented in the study are included in the article/supplementary material, further inquiries can be directed to the corresponding author.
